# Cognitive Healthy Aging in Mice: Boosting Memory by an Ergothioneine-Rich *Hericium erinaceus* Primordium Extract

**DOI:** 10.3390/biology12020196

**Published:** 2023-01-28

**Authors:** Elisa Roda, Fabrizio De Luca, Daniela Ratto, Erica Cecilia Priori, Elena Savino, Maria Grazia Bottone, Paola Rossi

**Affiliations:** 1 Laboratory of Clinical & Experimental Toxicology, Pavia Poison Centre, National Toxicology Information Centre, Toxicology Unit, Istituti Clinici Scientifici Maugeri IRCCS, 27100 Pavia, Italy; 2 Department of Biology and Biotechnology “L. Spallanzani”, University of Pavia, 27100 Pavia, Italy; 3 Department of Earth and Environmental Science, University of Pavia, 27100 Pavia, Italy

**Keywords:** aging, frailty, memory, medicinal mushroom supplementation, *Hericium erinaceus* primordium, ergothioneine, inflammation, oxidative stress, hippocampus, neuroprotection

## Abstract

**Simple Summary:**

With the increase in the geriatric population worldwide, the promotion of healthy aging arose as a key issue and, hence, the scientific community dedicates huge effort to counteract age-related impairments. Brain aging is a crucial risk factor for several neurodegenerative disorders and dementia. One of the most affected cognitive functions is recognition memory. Inflammation and oxidative stress play a key role in pathogenesis of cognitive impairments, and a link exists between frailty, oxidative stress, and *inflammaging*. Medicinal mushrooms represent a source to develop new therapeutic strategy, and among them *Hericium erinaceus* (He) displays several actions ranging from boosting immune system to fighting senescence, due to its active ingredients/metabolites. Among these, Ergothioneine (ERGO) is known as the longevity vitamin. Currently, when monitoring physiological aging in mice, we demonstrated the selective, preventive and neuroprotective effect of an ERGO-rich He primordium extract in hippocampus, preventing recognition memory decline during aging, decreasing key markers of inflammation and oxidative stress, and increasing the expression of glutamate receptors, which are crucially involved in glutamatergic neurotransmission.

**Abstract:**

Brain aging is a crucial risk factor for several neurodegenerative disorders and dementia. The most affected cognitive function is memory, worsening early during aging. Inflammation and oxidative stress are known to have a role in pathogenesis of cognitive impairments, and a link exists between aging/frailty and immunosenescence/*inflammaging*. Based on anti-aging properties, medicinal mushrooms represent a source to develop medicines and functional foods. In particular, *Hericium erinaceus* (He) displays several actions ranging from boosting the immune system to fighting senescence, due to its active ingredients/metabolites. Among these, Ergothioneine (ERGO) is known as the longevity vitamin. Currently, we demonstrated the efficacy of an ERGO-rich He primordium extract (He2) in preventing cognitive decline in a murine model of aging. We focused on recognition memory deterioration during aging, monitored through spontaneous behavioral tests assessing both memory components and frailty index. A parallel significant decrease in key markers of inflammation and oxidative stress, i.e., IL6, TGFβ1, GFAP, Nrf2, SOD1, COX2, NOS2, was revealed in the hippocampus by immunohistochemistry, accompanied by an enhancement of NMDAR1and mGluR2, crucially involved in glutamatergic neurotransmission. In summary, we disclosed a selective, preventive and neuroprotective effect of He2 on aged hippocampus, both on recognition memory as well on inflammation/oxidative stress/glutamate receptors expression.

## 1. Introduction

Aging is a process characterized by progressive frailty development in animals and humans. With the increase in the geriatric population worldwide, the promotion of healthy aging arose as a key issue. In particular, novel studies focus on therapeutic strategies with the goal to identify natural substances that may prevent, or even revert, aging-induced adverse effects.

Among cognitive functions known to worsen during brain aging, the most affected cognitive ability is episodic memory, declining early over time and displaying various severity extent [1,2,3]. Among episodic memory, recognition memory refers to the capability to discriminate a stimulus or an environment as familiar or novel. Recognizing objects, people, or environments previously encountered is a vital cognitive function in animals. Indeed, the recognition memory is considered one of the essential features of human and mammalian personalities [4]. Brain aging could be associated with cognitive impairment, motor disorders and emotive disturbances, being these alterations provoked by different morphological and functional changes involving the brain. Specifically, cellular, biochemical and molecular studies showed the imperative role played by inflammation and oxidative stress in the pathogenesis of cognitive impairments and age-associated neuronal diseases [5,6]. Undeniable evidence linked aging and frailty to both immunosenescence and chronic systemic inflammation, the alleged *inflammaging*, this latter mechanism being a distinctive feature of accelerated aging [7]. Chief molecules are involved in this process such as cytokines Interleukin-6 (IL6) and Transforming Growth Factor-beta1 (TGFβ1), inflammatory mediators known to participate in clinical and pathologic events during inflammatory diseases that are typically involved in the onset of chronic inflammation-induced fibrotic phenomena [8,9].

It is well established that glial cells are essentially involved during injury and neurodegenerative processes in the CNS. In particular, glial fibrillary acidic protein (GFAP) is usually employed as a marker of reactive gliosis associated with brain injury and CNS fibrotic events [10,11]. Experimental and epidemiological evidence demonstrated that aging brain is characterized by extensive gliosis in specific brain areas [12]. Therefore, the association between activated astrocytes/microglial cells with the release of soluble cytokines strongly suggests that inflammatory processes may play a critical task in the complicated pathophysiological interactions that occur during aging [13].

Other than inflammation and gliosis, mounting evidence emphasized the oxidative damage role in aging and age-associated cognitive and locomotor decline as the result of boosted reactive oxygen species (ROS) production and/or reduction in antioxidant scavengers [6,14,15,16]. Furthermore, in a loop manner, *inflammaging* onset enhances the vulnerability and responsiveness to stress-related molecules.

Concerning chief oxidative stress mediating factors, nuclear factor erythroid 2–related factor 2 (Nrf2) regulates the expression levels of several enzymes involved in oxidative stress and maintains cellular resistance to oxidants and the onset of the aging process [17]. Moreover, superoxide dismutase 1 (SOD1) is another principal protein involved in the enzymatic defenses against ROS production, participating in the maintenance of an appropriate free radicals’ level needed to preserve the physiological CNS functioning [18]. The overexpression of the inducible cyclooxygenase 2 (COX2) appears to play a dual role in the brain, acting as marker and effector of neural damage after brain injuries, in physiological or pathological aging [19,20].

The inducible form NOS2 is responsible for the generation of NO in different cells. Diverse studies confirmed that NO and pro-oxidants surplus are capable of triggering neuronal functional impairment and structural injury in some brain areas [21,22].

Glutamatergic pathway is involved in learning, memory formation/storage and synaptic plasticity [23]. It is also well known that aging is associated with cognitive deficits/decline via diverse mechanisms, including the change/impairment of glutamatergic pathway [5,24] and also affecting receptors’ binding and density [25,26,27]. Changes in the expression of the ionotropic receptors N-methyl-D-aspartate receptor (NMDARs) have been previously described in rat and murine hippocampus during aging or disease [28,29]. The G protein-coupled metabotropic glutamate receptors (mGluRs) are enriched in the hippocampal formation and interact physically with other proteins, including glutamate ionotropic receptors, to ensure the maintenance of cognitive performance. Changes in hippocampal mGluR expression levels were described in pathological cognitive aging [26,30,31].

*Hericium erinaceus* (He), also called Lion’s mane and Monkey Head Mushroom, is a palatable medicinal mushroom possessing many health properties including antioxidant, anti-inflammatory, antisenescence, neuroprotective and nootropic effects [32,33,34,35]. Ergothioneine (ERGO), a potent antioxidant molecule which gained the name of “longevity vitamin” [36,37], is often de novo synthesized in several fungi species.

In frail elderly people a negative correlation exists between plasma ERGO levels and cognitive decline. In particular, patients suffering from dementia have impaired cognitive ability correlated with declining plasma ERGO levels [38]. Scientists hypothesized that plasma ERGO levels could be a valuable tool, not only to early diagnose but also to provide effective therapeutic interventions able to mitigate cognitive decline. Recently, a significant enhancement of object recognition memory, determined by both NOR and OL tasks, was reported after oral administration of the food-derived ERGO in mice where an increased ERGO concentration was measured both in plasma and hippocampus level [39].

In previous recent papers we reported the presence of ERGO and other nootropic metabolites, in different strains of Italian *Hericium erinaceus*, highlighting their advantageous effects on both cognitive and locomotor performances in a murine model of physiological aging [7,40,41]. Lately, we encountered an *H. erinaceus* primordium that showed a peculiar high content of ergo but lacked both hericenones and erinacines [6,40]. Therefore, we seized the opportunity to explore the ergo-rich primordium preventive action on hippocampus during aging. In the present study a multi-tiered methodology allowed examination of potential neuroprotective effects with power to prevent cognitive decline during aging in mice. In particular, the experiments focused on: (i) monitoring spontaneous behavioral tests to gauge both components of recognition memory (i.e., knowledge/familiarity and remember/recollection), and the frailty index; and then (ii) investigating *inflammaging*, oxidative stress, gliosis and glutamatergic receptors expression in the hippocampus, appraising specific biomarkers. This study will give important information about the role of those mechanisms in this CNS area and on the selective protective effect of the ergothioneine rich primordium extract.

## 2. Materials and Methods

### 2.1. Animals

Fifteen pathogen-free C57BL-6J wild-type male mice were purchased from Charles River Italia, Calco, Italy. Mice were maintained in 12 h light/dark cycle in the Animal Facility of the University of Pavia at controlled temperature (21 ± 2 °C) and humidity (50 ± 10%). Water and food were provided *ad libitum*. All the mice were acclimatized for at least one month before starting the study.

All experiments were carried out in compliance with the guidelines laid out by the Ethics Committee of Pavia University for animal welfare (Ministry of Health, License number 774/2016-PR), and in accordance with the European Council Directive 2010/63/EU on the care and use of laboratory animals.

### 2.2. Experimental Plan Schedule

**Figure 1:**
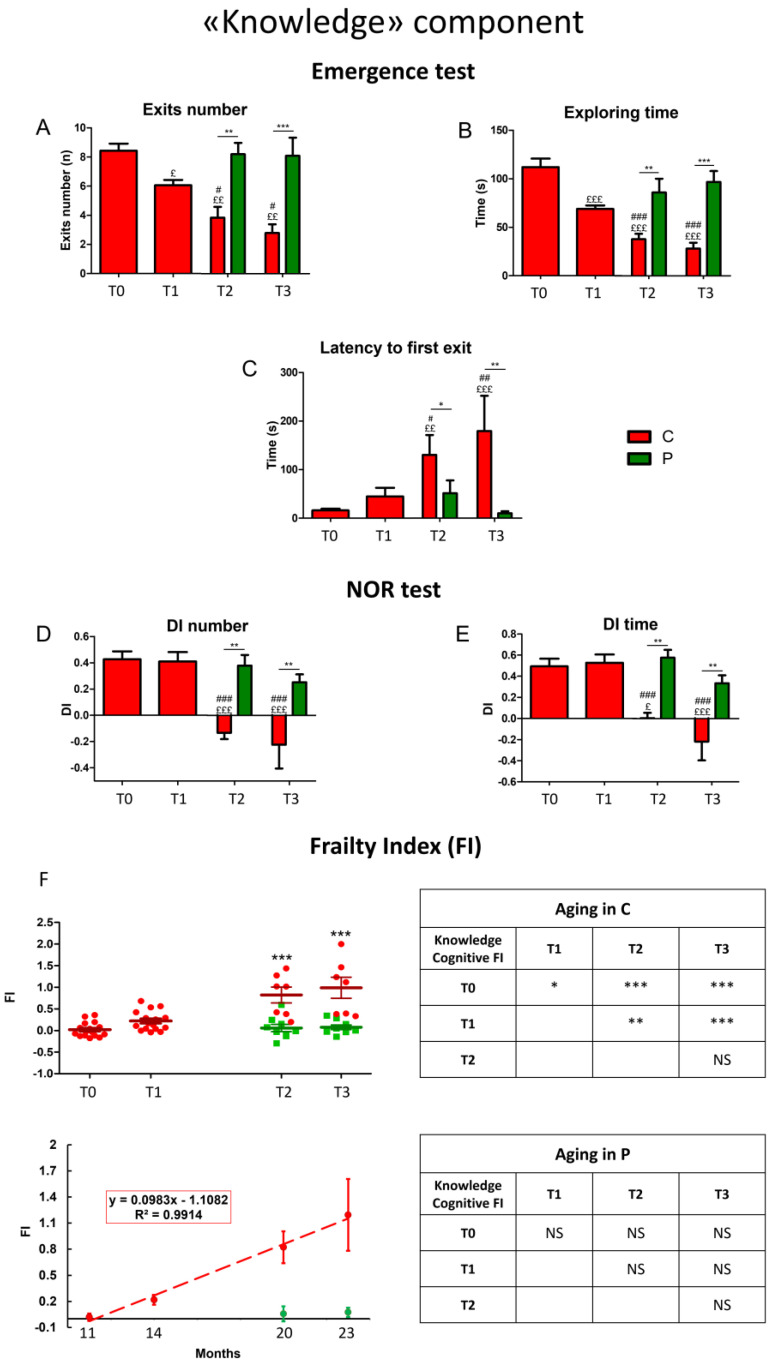
Physiological decline of the “Knowledge” component of recognition memory during aging in control mice and neuroprotection by He2 primordium extract in supplemented mice. For each panel, control (C) animals are represented with red bars, dots, and histograms, whereas supplemented (P) mice are symbolized by green bars, dots, and histograms. Panel (**A**−**C**) refer to Emergence test: (**A**) exits number, (**B**) exploring time, (**C**) 1st latency to firs exit. Panel (**D**,**E**) describe NOR test: (**D**) discrimination index (DI) of number of approaches, (**E**) DI of time of approaches. Panel (**F**): scatter plot showing integrated FIs for individual C and P mice (**left upright**); linear least-square regression of experimental points averaged data (**left downright**). Separated tables on the right display statistical results about aging effect in C (**upright**) and P (**downright**) animals. Statistically significant data: *p* < 0.05 (£, #, *); *p* < 0.01 (££, ##, **); *p* < 0.001 (£££, ###, ***).

In vivo experiments were performed at four different animal ages (corresponding to specifically reported experimental times): 11- and 14-months old (T0 and T1, respectively, occurring during adulthood phase); 20- and 23-months old (T2 and T3, respectively, taking place during senescence). At T3, tissue sampling and immunohistochemistry experiments were carried out. An 8-month lasting *H. erinaceus* oral supplementation was performed. Specifically, starting from 15 months of age, nine randomized mice were oral supplemented with a drink made with *H. erinaceus* (strain 2) primordium (called He2) ethanol extract solubilized in water (group I, namely P mice). The remaining six mice did not receive any supplementation (group II, namely C animals). The selected final dose of 1 mg of primordium/day was chosen to mimic the human oral supplementation (about 1 g/day). Hence, He2 primordium supplementation began during adulthood and continued until sacrifice at T3, during senescence. For experimental details see Figure 1.

Scientists conducting experimental procedures, i.e., spontaneous behavioral tests, immunohistochemistry, and statistical analyses, were blinded to the experimental condition.

### 2.3. Behavioral Tests and Cognitive Frailty Index

Spontaneous behavioral tests were performed to explore cognitive performances during aging. The choice of the behavioral test has been based on two main needs: (i) avoiding excessive animal handling; and (ii) translating the experimental results to human clinical settings. The emergence test and NOR tasks used in mice for studying the “Knowledge component” of the recognition memory are similar to the “Stenberg Item Recognition paradigm” conventionally utilized in clinical practice. Furthermore, OL and Y maze tasks used in mice to investigate the Remember component of the recognition memory are like the “Four mountains test and Image-location memory task” employed by hospital clinicians.

Animals’ performances were measured by using SMART video tracking system (2 Biological Instruments, Besozzo, Varese, Italy) and Sony CCD color video camera (PAL). At different selected experimental times all mice performed different spontaneous behavioral tests for investigating both components of spontaneous recognition memory: knowledge/familiarity and remember/recollection.

Emergence and Novel Object Recognition tasks (NOR) evaluated the knowledge component, whereas Y maze and Object location tasks (OL) assessed the remember component.

All these tests were conducted as previously described [42,43]. For each test we selected specific parameters that decline during aging (Table 1).

For each selected parameter we obtained the corresponding cognitive frailty index (FI), by using the following formula [41]:

FI = (Value-Mean Value at T0)/(SD at T0) × 0.25

For each test we averaged the FIs for each parameter and obtained the Fi score for the specific spontaneous test. Then, we obtained the averaged cognitive FI for all tests and the FI for each component of the recognition memory. Finally, by averaging the knowledge and remember recognition memory FIs we obtained a global cognitive FI.

### 2.4. H. erinaceus (He2) Primordium: Isolation/Cultivation, Extraction Procedures and Analytical Determinations

As previously described, the He2 strain was isolated from a wildtype sporophore collected in Italy in 2018 [44]. The sporophore was aseptically cut into small pieces (about 1 mm^3^) that were inoculated in Petri dishes containing 2% malt extract agar (MEA). The isolated strain is preserved in the MicUNIPV, the Fungal Research Culture Collection of University of Pavia, Italy. Through isolation in pure culture, mycelium was for obtaining the sporophore, as previously reported [41]. The primordium developed at the initial stages of growth; it was collected, analyzed, and used for the murine supplementation.

Concerning extraction procedure and analytical measurements, they were carried out as previously reported in detail [6]. Briefly, 1 g of dried He2 primordium was blended with ethanol 70% (10 mL) and then processed as described in literature [41,45]. HPLC-UV-ESI/MS was used to identify and measure the ERGO amount in He2 primordium extract by comparison with ERGO calibration curve (L-(+)-Ergothioneine (497-30-3, TETRAHEDRON, Paris, France) as standard (for details, see [6]). The ERGO calibration curve was constructed by injecting five different concentrations of standard mixture solutions (10, 70, 150, 350 mg/L, analyzed in triplicate).

### 2.5. Necropsy, Tissue Sampling and Immunohistochemical Analyses

#### 2.5.1. Brain Specimens’ Preparation

At T3, 23-months-old mice were deeply anesthetized by isoflurane inhalation (Aldrich, Milwaukee, WI, USA) before sacrifice. Brains were immediately excised as previously described [7], washed in 0.9% NaCl, and fixed by immersion for 48 h at room temperature in 4% paraformaldehyde in 0.1 M phosphate buffer (pH 7.4). Tissues were then dehydrated in absolute ethanol, followed by acetone, and finally embedded in Paraplast X-TRA (Sigma Aldrich, Milan, Italy). Eight micrometer-thick sections of brains were cut in the coronal plane and collected on silane-coated slides. After employing Hematoxylin and Eosin (H&E) histological staining [46], precise sections were chosen to study the hippocampus.

#### 2.5.2. Immunohistochemistry and Bright-Field Microscopy

To explore expression and distribution of specific molecules, immunohistochemical experiments were performed as previously reported [7] using commercial antibodies on murine brain specimens. Interleukin-6 (IL6), Transforming Growth Factor-beta1 (TGFβ1), glial fibrillary acidic protein (GFAP), nuclear factor erythroid 2–related factor 2 (Nrf2), superoxide dismutase 1 (SOD1), cyclooxygenase 2 (COX2), nitric oxide synthase 2 (NOS2), metabotropic glutamate receptor 2 (mGluR2) and ionotropic N-methyl-D-aspartate (NMDA) receptor 1 (NMDAR1). Coronal brain sections from C and P mice were incubated overnight at RT with PBS-diluted monoclonal and polyclonal primary antibodies (Table 2). IL6, TGFβ1 and GFAP were evaluated as specific markers of inflammation and reactive gliosis, respectively; Nrf2, SOD1, COX2 and NOS2 were assessed being essentially involved in oxidative stress cascade [6,47,48,49]; metabotropic glutamate receptors-2 (mGluR2) and N-methyl-d-aspartate (NMDA) receptor-1 (NMDAR1) were considered for their crucial role in glutamatergic pathway [50,51]. To reveal the antigen/antibody interaction sites, proper biotinylated secondary antibodies (Table 2) and an avidin biotinylated horseradish peroxidase complex (Vector Laboratories, Burlingame, CA, USA) were used. The 3,3′-diaminobenzidine tetrahydrochloride peroxidase substrate (Sigma, St. Louis, MO, USA) was employed as the chromogen, and the nuclear counterstaining was achieved by using Carazzi’s Hematoxylin. Then, the sections were dehydrated in ethanol, cleared in xylene, and mounted in Eukitt (Kindler, Freiburg, Germany). As negative control some sections were incubated with PBS only, in the absence of the primary antibodies, revealing a complete lack of immunoreactivity.

#### 2.5.3. Immunohistochemical Evaluations

Sections were observed in brightfield microscopy using an Olympus BX51 optical microscope (model BX51TF, Olympus Italia S.r.l, Segrate, Italy) and images were acquired with an Olympus CAMEDIA C4040ZOOM camera. For each assessed marker, five slides (about 20 sections) per mouse were examined. In both experimental groups’ hippocampal specimens with diverse immunolabeling extents were considered. For each immunohistochemical reaction the most representative figures were selected and are shown. Immunohistochemical labeling degree was measured on acquired digitized section images under exposure time avoiding any pixel saturation effect. Immunoreactive cell density (number of immunopositive cells/area in mm^2^) and labeling intensity were calculated utilizing densitometric analysis (Image-J 1.48i; NIH, Bethesda, MA, USA), as previously described [7]. Briefly, the immunocytochemical intensity, namely OD, was evaluated in three randomized images/section per five slides/animal from each experimental group. Data were recorded on Microsoft Office Excel Software spreadsheets and the analysis was achieved using the ImageJ software.

### 2.6. Statistics

Data were reported as mean standard error of the mean (SEM). We performed Bartlett and Shapiro–Wilk Tests to establish and confirm the normality of parameters. Concerning behavioral tests, One-way Anova for repeated measures was used for investigating the aging effect (£ vs. 11 months (T0), # vs. 14 months; $ vs. 20 months), whereas two-way Anova was used to compare C and P groups. The differences were considered statistically significant for *p* < 0.05 (£, #, $, *), *p* < 0.01 (££, ##, $$, **), and *p* < 0.001 (£££, ###, $$$, ***).

The statistical analysis for immunohistochemical evaluations was carried out using an unpaired Student’s *t*-test. The differences were considered statistically significant for *p* < 0.05 (*), *p* < 0.01 (**), and *p* < 0.001 (***).

All statistical analyses were performed with GraphPad Prism 8.0 software (GraphPad Software Inc., La Jolla, CA, USA).

## 3. Results

As previously reported, He2 primordium extract, characterized by HPLC-UV-ESI/MS, contains 1.3 ± 0.57 mg/g ERGO [6], being this amount three times higher than that measured in both mycelium (0.58 mg/g) and sporophore (0.34 mg/g) of the strain He1 [7]. Furthermore, primordium extract does not contain the nootropic metabolites hericenones or erinacine [6].

### 3.1. Cognitive Outcomes

#### 3.1.1. He2 Preventive Effect on Aging Decline of the “Knowledge” Memory Component

For a longitudinal study of the “knowledge” component during aging, we measured selected parameters (see Table 1 in Section 2) in the emergence and NOR tests at chosen experimental times: 11 (T0), 14 (T1), 20 (T2), and 23 (T3) months (Figure 1) in both C and He2 supplemented (namely P) mice.

In C animals, all parameters recorded during Emergence task, such as exits number, exploring time, and latency to first exit (Figure 1A–C, see red bars and dots) worsened over time, from T0 to T3. Notably, after T1 the oral supplementation with He2 primordium extract significantly improved all recorded parameters until T3. Indeed, P group showed similar values for all recorded parameters in adulthood (T0) and in senescence phases (T2 and T3) (Figure 1A–C, see green bars and dots).

In NOR test the discrimination index related to both number and time of approaches (Figure 1D,E, respectively), deteriorated after T2 in C mice, reaching negative values at T2 and T3. Remarkably, after T1 the He2 supplementation significantly inhibited the cognitive deterioration in wild-type mice both at T2 and T3. Indeed, P mice displayed similar values for all recorded parameters during the entire investigated life span (from adulthood to senescence phases) and, further, these values were significantly different from those measured in C animals during senescence, i.e., T3 and T4.

Finally, we calculated the cognitive FI for each parameter in the emergence and in the NOR tests and we separately averaged the FIs for the two tests. Then, averaging the two obtained FIs, we calculated the global, integrated cognitive FIs of the knowledge component of the recognition memory (Figure 1F).

The integrated knowledge cognitive FI significantly increased during aging in C animals (C, red dots) in a straight way, as demonstrated by linear least-square regression analysis (R^2^ = 0.99). Outstandingly, P mice (P, green dots) did not develop frailty hallmarks during aging, thus demonstrating the preventive effect of the oral supplementation with He2 primordium extract on this specific component of the recognition memory.

#### 3.1.2. He2 Preventive Effect on Aging Decline of the “Remember” Memory Component

For a longitudinal study of the “remember” component during aging we gauged selected parameters (see Table 1 in Section 2) in OL and Y maze tests at specific chosen experimental times: 11 (T0), 14 (T1), 20 (T2), and 23 (T3) months in C and He2 supplemented P mice.

**Figure 2:**
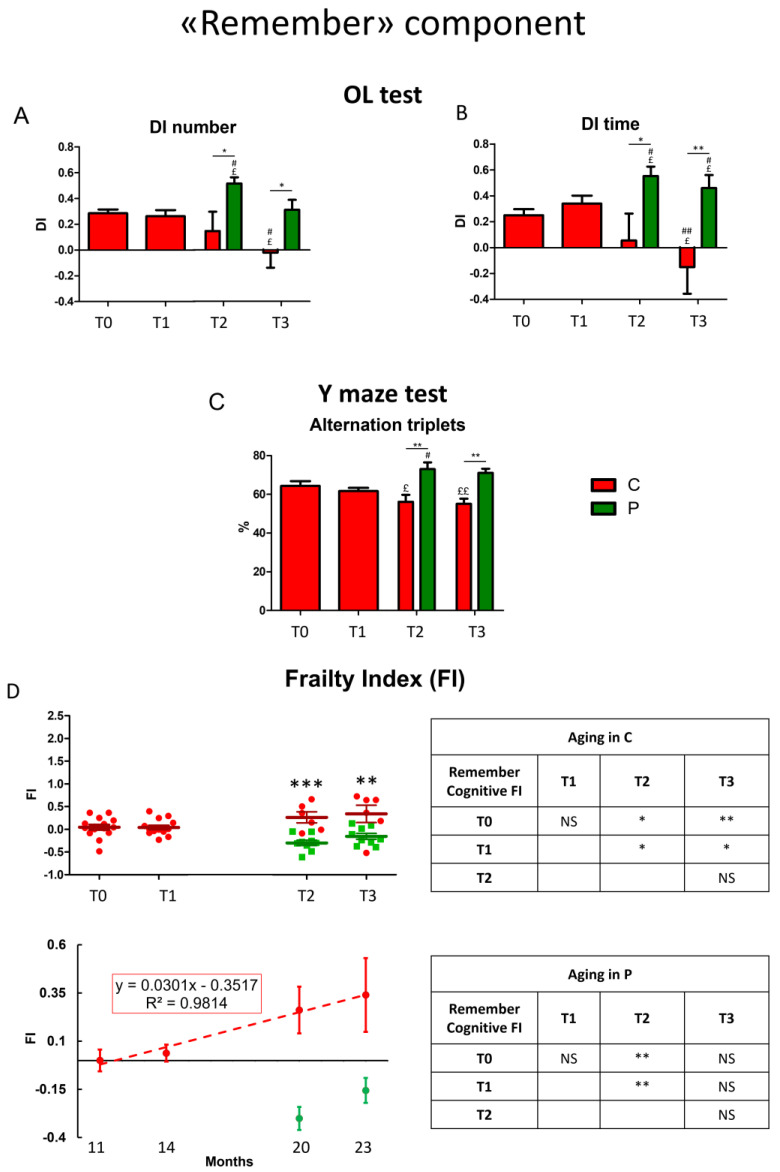
Physiological decline of the “Remember” component of recognition memory during aging in control mice and neuroprotection by He2 primordium extract in supplemented mice. For each panel, control (C) animals are represented with red bars, dots, and histograms, while supplemented (P) mice are symbolized by green bars, dots, and histograms. Panel (**A**,**B**) describe OL test: discrimination index (DI) of the number (**A**) and time of approaches (**B**). Panel (**C**) is related to Y maze task (alternation triplets %). Panel (**D**) display Remember cognitive Frailty Index (FI): scatter plot showing integrated FIs for individual C and P mice (**left upright**); linear least-square regression of experimental points averaged data (**left downright**). On the right, separated tables report statistical data concerning age-related effect in C (**upright**) and P (**downright**) mice. Statistically significant data: *p* < 0.05 (£, #, *); *p* < 0.01 (££, ##, **); *p* < 0.001 (***).

During physiological aging, in OL test the discrimination index related to both number and time of approaches worsened after T2 in C mice (Figure 2A,B, red bars). Differently, after T1 the He2 primordium extract oral supplementation prevented the cognitive decline hindering the deterioration of the discrimination index during the senescence phases (Figure 2A,B, green bars). Furthermore, in P mice the discrimination index of both selected parameters, measured at T2 and T3, was significantly higher compared to that gauged in C animals, suggesting not only a prevention of cognitive decline during aging but also an He2-induced “gain of function” of this memory component.

The cognitive parameters evaluated during Y maze task, the alternation triplets’ percentage, appreciably decreased during the senescence phases, both at T2 and T3 (Figure 2C). Remarkably, after T1 the He2 primordium extract oral supplementation substantially prevented this cognitive deterioration, and, furthermore, increased this cognitive parameter in a significant manner compared to C mice. In accordance with previously described data, even in this case we assumed a gain of function in the remember memory component adjuvated by He2 primordium oral supplementation.

Next, we calculated the cognitive FI for each parameter and the obtained value for each test was averaged. Finally, we averaged the two-frailty index for OL and Y maze test in an integrated, global FI of the remember component of recognition declarative memory (Figure 2D). Similar to the situation above described for knowledge memory component, remember cognitive FI significantly increased during aging in C animals (C, red dots) in a straight way, as demonstrated by linear least-square regression analysis (R^2^ = 0.98). Notably, in P mice (P, green dots) after supplementation, the remember component reached a value higher than that measured in young animals suggesting a boosting effect or a “gain of function” of this component despite animal age.

### 3.2. Histological and Immunohistochemical Data

All reactions were conducted on coronal brain sections from both aged non-supplemented, i.e., controls (C animals), and aged He2 primordium-treated mice (P group) at T3 (23-month-old animals), focusing on the hippocampus being crucial in recognition and spatial memory [52,53].

All examinations focused on the dentate gyrus (DG), and the Ammon’s horn region (including CA subdivisions) of the hippocampus where oxidative stress and inflammation were predominantly localized.

#### 3.2.1. He2 Supplementation Preserves Hippocampus Healthy Cytoarchitecture

**Figure 3:**
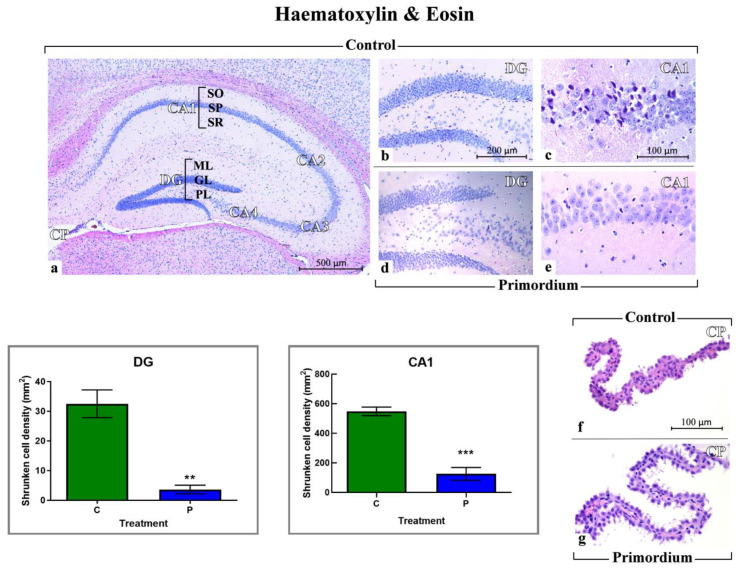
Histological characterization by H&E staining. Representative brain sections, showing the well-preserved physiological hippocampal cytoarchitecture both in non-supplemented controls (**a**–**d**) and P (**d**,**e**) aged mice. (**a**): low magnification micrograph shows the whole hippocampus, formed of cornu Ammonis (CA) and dentate gyrus (DG). CA is further partitioned into: CA1, CA2, CA3 and CA4. The choroid plexus (CP) of the lateral ventricle can be also observed. (**b**,**d**): higher magnifications of the DG area revealing well-defined three layers: molecular layer (ML), granule cell layer (GL) and pleomorphic layers (PL). (**c**,**e**): higher magnifications of the CA1 region, showing the typical three layered-structure. Outer polymorphic layer, i.e., *Stratum oriens* (SO); middle pyramidal cell layer, namely *Stratum pyramidale* (SP); inner molecular layer, i.e., *Stratum radiatum* (SR). (**f**,**g**): choroid plexus (CP) in C and P mice, respectively. (**f**): evident structural alterations were observable, with ependymal cells displaying cilia reduction. Light microscopy magnification: 40× (**a**), 200× (**b**,**d**), 400× (**c**,**e**–**g**). Scale bars: 500 µM (**a**); 200 µM (**b**,**d**); 100 µM (**c**,**e**–**g**). **Lower left panels**: Histograms showing the quantitative assessment of shrunken cell density in DG and CA1 region of Ammon’s horn. *p* values calculated by unpaired Student’s *t*-test: *p* < 0.01 (**), and *p* < 0.001 (***).

H&E staining was employed to estimate the potential occurrence of age- and/or He2-related changes in hippocampus cytoarchitecture in aged C and P mice. Representative pictures obtained by H&E in C (Figure 3a–c,f) and P (Figure 3d,e,g) mice are illustrated in Figure 3. The physiological gross morphology of the whole hippocampus was preserved in both experimental groups (Figure 3a–d). In particular, in C and P mice high-magnification micrographs of the DG revealed well-defined three layers, namely molecular layer (ML), granule cell layer (GL) and pleomorphic layer (PL). Concerning the CA1, the typical three layered-structure was observed, characterized by the presence of the outer polymorphic layer, namely *Stratum oriens* (SO), the middle pyramidal cell layer, namely *Stratum pyramidale* (SP) and the inner molecular layer, namely *Stratum radiatum* (SR).

Nonetheless, distinct structural alteration of the choroid plexus was revealed in control mice only (Figure 3f), with ependymal (choroid epithelial) cells displaying an evident cilia reduction. Additionally, comparing C and P animals, a significant increase in shrunken cell density was measured both in DG and particularly in the CA1 region of Ammon’s horn in the C mice only (Figure 3).

#### 3.2.2. He2 Supplement Decreases Inflammaging

**Figure 4:**
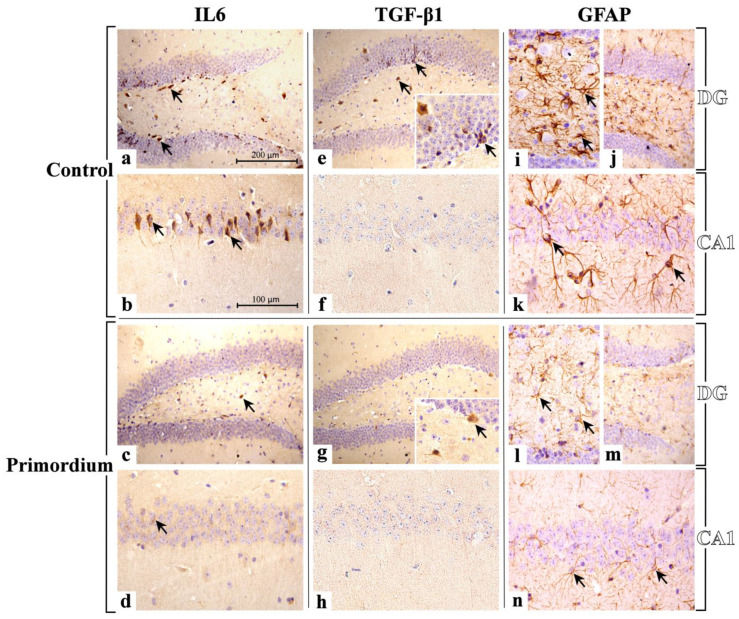
Immunostaining patterns of IL6 (**a**–**d**), TGFβ1 (**e**–**h**) and GFAP (**i**–**n**) expression in C animals (**a**,**b**,**e**,**f**,**i**–**k**) and P (**c**,**d**,**g**,**h**,**l**–**n**) mice. **IL6**: an evident IL6 immunolabelling is observable both in DG and CA1 of C mice ((**a**,**b**), respectively), showing several markedly immunopositive neurons (arrows). Diversely, in supplemented P mice, a pale IL6-immunopositivity is observed in both areas ((**c**,**d**) for DG and CA, respectively), where rare immunolabelled cells are discernible (arrows). **TGFβ1**: a strong immunoreactivity for TGFβ1 is observed mainly in DG of C mice (**e**), where different clusters of immunopositive neurons are visible (arrows). Sporadic immunopositive cells (arrows) are observed in the DG of P animals (**g**). Differently, any immunostaining is observable in CA1 region of both C and P mice ((**f**,**h**), respectively). **GFAP**: a widespread GFAP immunolabelling is distributed both in DG and CA1 of C (**i**–**k**) and P (**l**–**n**) mice. In particular, in C animals, a carpet of GFAP-immunopositive astrocytes is clearly evident, showing thickened and intensely stained soma and prolongations (arrows). Light microscopy magnification: 200× (**a**,**c**,**e**,**g**,**j**,**m**); 400× (**b**,**d**,**f**,**h**,**i**,**k**,**l**,**n**); 600× (Insert in **e**,**g**). Scale bars: 200 µM (**a**,**c**,**e**,**g**,**j**,**m**); 100 µM (**b**,**d**,**f**,**h**,**i**,**k**,**l**,**n**).

**Figure 5:**
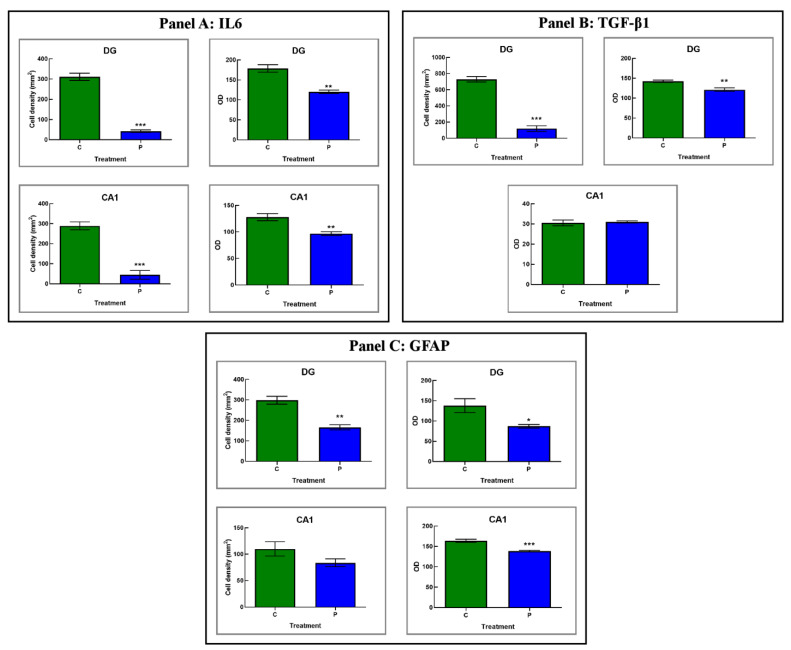
Panels (**A**–**C**): Histograms showing the quantitative analysis of IL6-, TGFβ1- and GFAP-immunopositive cell density and OD, respectively, as determined in DG and CA1 region of C and P mice. *p* values calculated by unpaired Student’s *t*-test: *p* < 0.05 (*), *p* < 0.01 (**), and *p* < 0.001 (***).

In the current study we immunohistochemically assessed the presence/distribution of specific molecules, i.e., IL6, TGFβ1, and GFAP, as typical indicators of the inflammatory pathway (Figure 4 and Figure 5 and Table 3). For all investigated markers, in both experimental groups, i.e., C and P mice, the immunopositivity was mainly localized in the DG and CA1 region of Ammon’s horn.

Concerning IL6, in the present study deeply IL6-immunostained neurons were clearly observed in the DG (mainly located among the round to oval granule cell bodies aggregation in the GL) of C mice only. Notably, several immunopositive neurons localized nearby the sub-granular zone (SGZ) were also detected. Furthermore, in the same animals, markedly IL6-immunoreactive neurons, possibly mossy cells, were revealed in the PL.

Concerning the evaluation of CA1, the SP exhibited several IL6-immunopositive pyramidal neurons showing closely packed cell somas regularly arranged in 4 to 5 strings, with evidently immunomarked prolongations.

The subsequent quantitative analyses revealed an extremely significant reduction in IL6-immunopositive cell density measured in both DG and CA1 in P mice only. A significant reduction in IL6-immunopositive cells OD was determined in both DG and CA1 of P mice, compared to controls (Table 3 and Figure 5).

TGFβ1 immunostaining was revealed in C mice only, mainly localized in GL and PL of DG, whereas any immunostaining was detected in C and P mice in CA1 area (Figure 4 and Figure 5 and Table 3). In detail, different clusters of immunopositive neurons were observed, some of which were characterized by a large soma (mainly observed in PL); other smaller ones assembled in orderly chains, typically arranged in the thickness of GL. The quantitative assessment confirmed an extremely significant reduction in immunoreactive cell density in P mice compared to C animals. Furthermore, a significant reduction in TGFβ1-immunopositive cells OD was determined in the DG of P mice only (Table 3). As regards the CA1 area, any significant difference in TGFβ1-immunoreactive cell OD was measured comparing P and C mice (Table 3).

Herewith, the localization of GFAP showed a widespread distribution both in DG and CA1 of P and C mice. In particular, in these latter animals a carpet of GFAP-positive astrocytes was observed, showing thickened and intensely stained soma and prolongations mainly located in the PL of DG and in the whole thickness of CA1.

The subsequent quantitative study confirmed a significant lessening of immunopositive glial cells for GFAP in the DG of P mice compared to C animals. Similarly, a significant reduction in GFAP-immunopositive cells OD was measured in the DG of P mice compared to controls. Likewise, a slight decrease in immunopositive glial cell density was measured in CA1 region of P mice compared to C. Similarly, a significant decrease in GFAP immunopositive cells’ OD was measured in the CA1 area of P animals only (Table 3).

#### 3.2.3. He2 Supplement Diminished Age-Related ROS Levels

**Figure 6:**
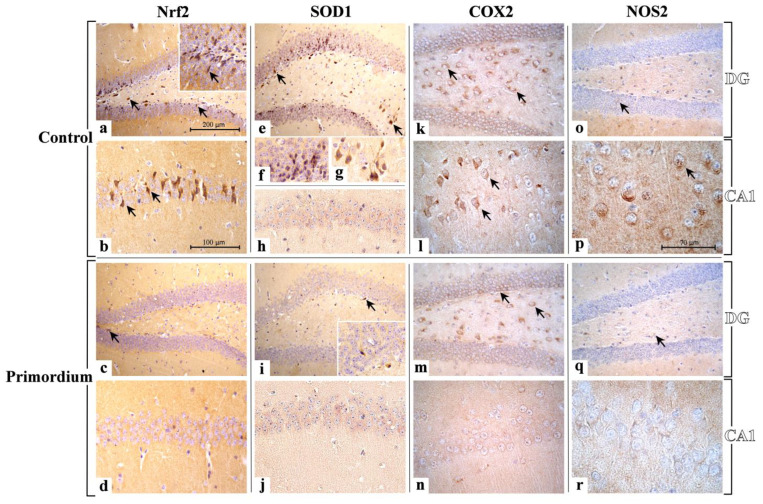
Representative micrographs showing Nrf2, SOD1, COX2 and NOS2 immunohistochemical expression in DG and CA1 area from non-supplemented C animals ((**a**,**b**,**e**–**h**,**k**,**l**,**o**,**p**), respectively) and P ((**c**,**d**,**i**,**j**,**m**,**n**,**q**,**r**), respectively) mice. **Nrf2:** the heaviest immunopositivity is detectable almost exclusively in C mice (**a**,**b**), mainly in the DG region (**a**), with several markedly immunopositive cells closed to the SGZ (arrows), and also in the PL, where bigger immunoreactive neurons are observable (arrows). Nrf2 antigen is also overexpressed in pyramidal neurons of CA1 region (**b**). Diversely, immunopositive cells are rarely detectable in P mice (**c**,**d**), only in the DG (**c**) while the CA1 lacks immunoreactivity (**d**). **SOD1**: heavily immunopositive cells, localized both in the width of the GL as well as nearby the SGZ, are clearly visible in the DG (**e**,**f**) of C mice (**e**–**h**). Also, strongly immunomarked neurons appear evident in the PL (**g**). Few immunopositive cells are observed in DG of P mice (**i**). A widespread lack of SOD1-immunoreactivity is detectable in the CA1 region both in C and P animals ((**h**,**j**), respectively). **COX2**: the strongest immunopositivity was detected in the DG of both C and P animals ((**k**,**m**), respectively), showing several immunoreactive neurons in the PL (arrows). Several strongly immunomarked pyramidal neurons are observable in the CA1 area of P mice (**l**), while a complete absence of immunoreactivity is evident in C animals (**n**). **NOS2**: a scarce NOS-immunopositivity was observed in the DG of both C and P mice (**o**,**q**, respectively), where palely immunoreactive cells are visible in the PL (arrows). An evident immunoreactivity is identifiable in neurons (arrows) located in the CA1 region of C mice (**p**), while a complete absence of immunopositivity is visible in P animals (**r**). Light microscopy magnification: 200× (**a**,**c**,**e**,**i**,**k**,**m**,**o**,**q**); 400× (**b**,**d**,**h**,**j**,**l**,**n**); 600× ((**f**,**g**,**p**,**r**) and insert in (**a**,**i**)). Scale bars: 200 µM (**a**,**c**,**e**,**i**,**k**,**m**,**o**,**q**); 100 µM (**b**,**d**,**f**,**g**,**h**,**j**,**l**,**n**); 70 µM (**p**,**r**).

**Figure 7:**
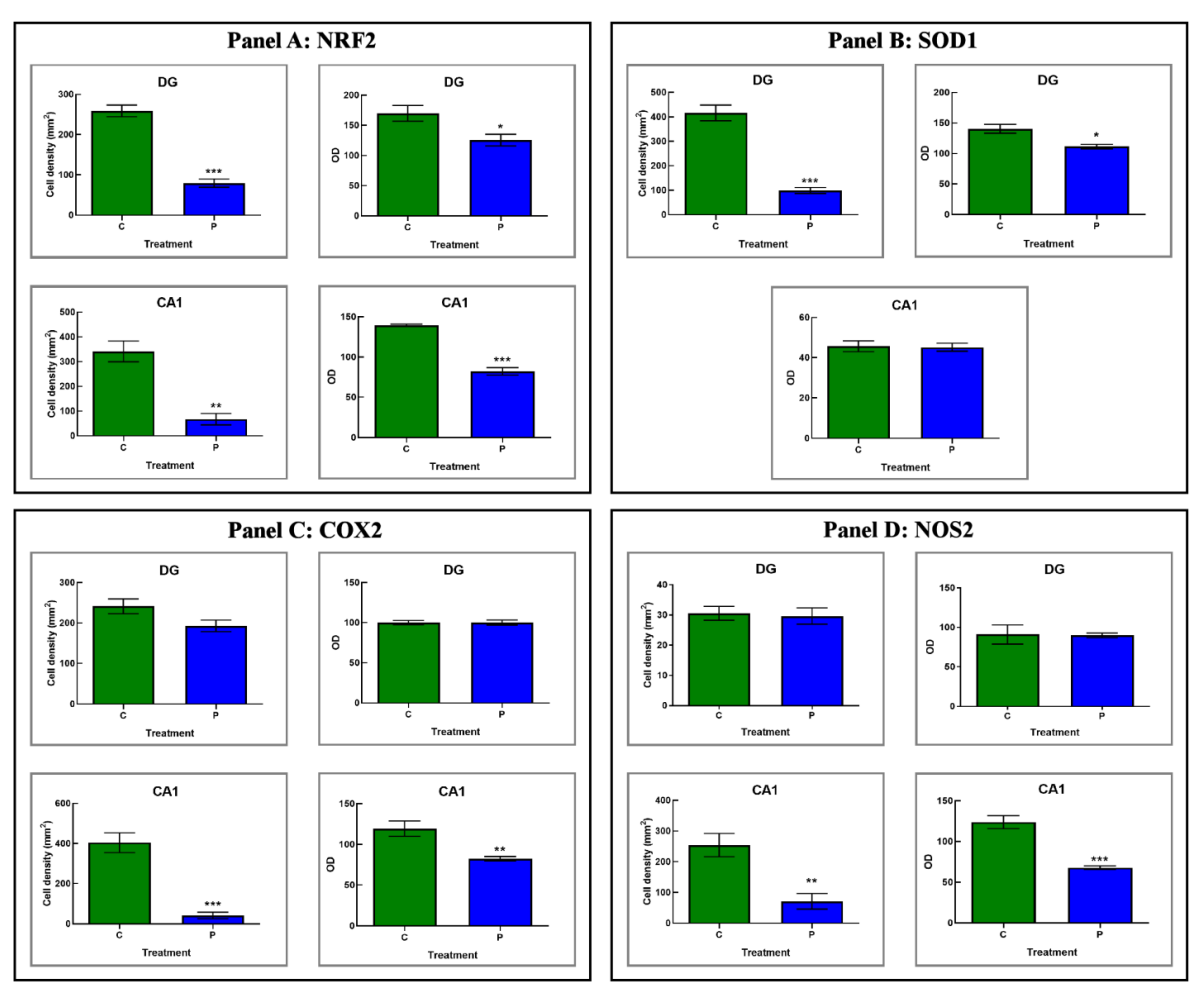
Panels (**A**–**D**): Histograms showing the quantitative assessment of Nrf2-, SOD1-, COX2- and NOS2-immunopositive cell density and OD, respectively, measured in DG and CA1 region of C and P mice. *p* values calculated by unpaired Student’s *t*-test: *p* < 0.05 (*), *p* < 0.01 (**), and *p* < 0.001 (***).

In the current study we assessed, by light microscopy immunohistochemistry, the presence and distribution of crucial player of the oxidative stress pathway, i.e., Nrf2, SOD1, COX2 and NOS2 (Figure 6 and Figure 7 and Table 4). Like the above reported data concerning inflammation, the immunoreactivity for all these oxidative stress markers was mostly detected in the DG and CA1 region of Ammon’s horn in both experimental groups, namely C and P mice.

Currently, immunohistochemical reaction for **Nrf2** revealed the heaviest immunopositivity almost exclusively in C mice, mainly localized in the DG region, with several markedly immunopositive cells closed to the SGZ, showing condensed nuclei, and also in the PL, where bigger immunoreactive neurons, characterized by large somas, were observed (Figure 6). Notably, in the same experimental group only, namely C animals, Nrf2 antigen resulted also overexpressed in pyramidal neurons of CA1 region (Figure 6).

Accordingly, the quantitative analysis documented an extremely significant reduction in immunolabeled cell density in the DG of P mice compared to controls. Furthermore, a significant parallel decrease in immunopositive cells OD was observed in the same area comparing P and C mice (Figure 7 and Table 4).

With a similar trend, a very significant reduction in Nrf2-immunopositive cell density was detected in the CA1 area comparing P and C mice. Concerning Nrf2-immunoreactive cells OD, an extremely significant lessening was measured in the CA1 area of P mice compared to C animals (Figure 7 and Table 4).

Regarding SOD1, the expression pattern differed strikingly between the two experimental groups, namely C and P mice. Specifically, in C animals only, the DG was characterized by clusters of heavily immunopositive cells localized both in the width of the GL as well as nearby the SGZ. Moreover, in the same area, strongly immunomarked neurons appeared evident in the PL. Concerning the CA1 region, a widespread lack of immunoreactivity for SOD1 was revealed both in C and P animals (Figure 6).

Hence, the subsequent quantitative evaluation determined an extremely significant decrease in SOD1-immunolabeled cell density in the DG comparing P and C mice. Likewise, a significant reduction in SOD1-immunopositive cells OD was observed in the DG of P animals only (Figure 7 and Table 4). As regards the CA1 area, in line with the qualitative observation noticed in light microscopy any significant change in SOD1-immunoreactive cell OD was measured comparing P and C mice (Figure 7 and Table 4).

Presently, the cellular localization and distribution of COX2 revealed a similar immunostaining pattern in the DG of both experimental groups, namely C and P mice. In detail, the stronger immunopositivity was detected at cytoplasmic level in the neurons of PL (Figure 6). Concerning the CA1 area, the COX2-immunoreactivity greatly diverged between P and C mice. Specifically, several strongly immunomarked pyramidal neurons were observed in P mice only, while a complete absence of immunoreactivity was revealed in C animals (Figure 6).

The quantitative analysis documented only a slight reduction in COX2-immunopositive cell density in the DG comparing P and C mice (Figure 6 and Figure 7 and Table 4). Likewise, focusing on immunolabeled cells OD, any significant difference was measured in the DG comparing the two experimental groups (Table 4). Diversely, with regard to the CA1 area, an extremely significant reduction in COX2-immunolabeled cell density was detected in P mice compared to controls. Moreover, in the same supplemented animals, a very significant lessening of immunopositive cell OD was determined (Figure 7 and Table 4).

Concerning NOS2, the heaviest immunoreactivity was mainly identified in the cytoplasm of pyramidal neurons of the CA1 region of the Ammon’s horn in C mice only (Figure 6). Differently, concerning the DG, in both experimental groups, namely P and C animals, a pale NOS-immunopositivity was observed in rare cells of the PL while any immunopositivity was detected in the GL (Figure 6).

Interestingly, the quantitative evaluation revealed an immunostaining pattern trend comparable to that above reported for COX2. Specifically, only a slight non-significant reduction in NOS2-immunopositive cell density was calculated comparing the two experimental groups (Figure 7 and Table 4). Showing a similar trend, any significant difference was measured considering NOS2-immunolabeled cell OD both in P and C mice (Figure 7 and Table 4). Considering the CA1, a very significant reduction in NOS2-immunolabeled cell density was reported in P mice compared to controls. Likewise, an extremely significant reduction in NOS2-immunopositive cell OD was assessed comparing P and C animals (Figure 7 and Table 4).

### 3.3. He2 Supplement Improved Glutamate Neurotransmission in Aging

**Figure 8:**
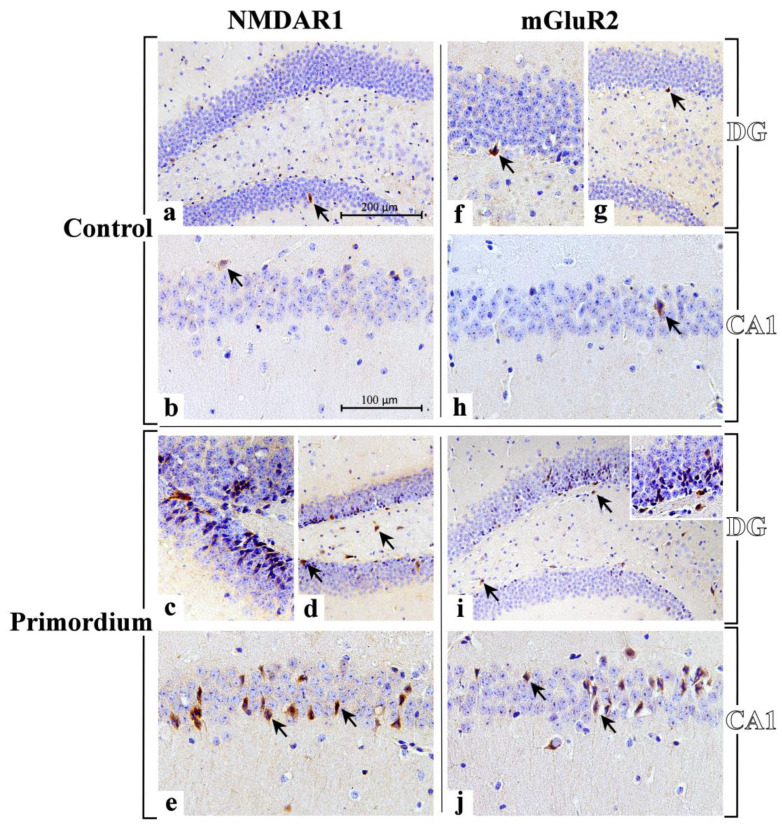
Immunohistochemical labelling for NMDAR1 and mGluR2 in DG and CA1 region from C animals ((**a**,**b**,**f**–**h**), respectively) and P ((**c**–**e**,**i**,**j**), respectively) mice. **NMDAR1**: sporadic immunolabelled cells (arrows) are observable both in DG (**a**) and CA1 region (**b**) of C animals. A marked immunopositivity is clearly evident both in the DG and CA1 area of P mice ((**c**–**e**), respectively). In the DG, clusters of heavy immunoreactive cells, arranged in well-ordered chains, are observable in the width of the GL (**c**), several immunolabelled cells are in the SGZ (**d**, arrows), and many immunoreactive neurons with large soma are detectable in the PL ((**d**), arrows). In the CA1 area several NMDAR1-immunopositive neurons are visible localized in the SP, often showing palely immunomarked tiny prolongations, deepening in the underneath SR ((**e**), arrows). **mGluR2**: a weak and sporadic immunoreactivity is observable both in DG (**f**,**g**) and CA1 region (**h**) of C animals, where rare immunolabelled cells are detected (arrows). A heavy immunopositivity is clearly evident both in the DG and CA1 area of P mice ((**i**,**j**), respectively). Some heavily immunoreactive cells are evident in the DG (**i**), principally localized in the width of the GL and close to the SGZ (arrows). Various immunomarked neurons are also noticeable in the PL ((**i**), arrows). Numerous immunopositive neurons are detectable in the SP of CA1, often characterized by immunoreactive prolongations, deepening beneath in the underlying SR (**j**). Light microscopy magnification: 200× (**a**,**d**,**g**,**i**); 400× (**b**,**e**,**h**,**j**); 600× ((**c**,**f**) and insert in (**i**)). Scale bars: 200 µM (**a**,**d**,**g**,**i**); 100 µM (**b**,**c**,**e**,**f**,**h**,**j**).

In the current study, immunohistochemical pictures obtained assessing specific markers of glutamatergic neurotransmission, i.e., NMDAR1 and mGluR2, molecules belonging to the two general receptors classes of glutamatergic pathway, are depicted in Figure 8. In particular, the localization of these two key molecules showed a widespread distribution in the DG and CA1 area of Ammon’s horn in both experimental groups, namely P and C mice, as formerly reported for all the other investigated markers.

**Figure 9:**
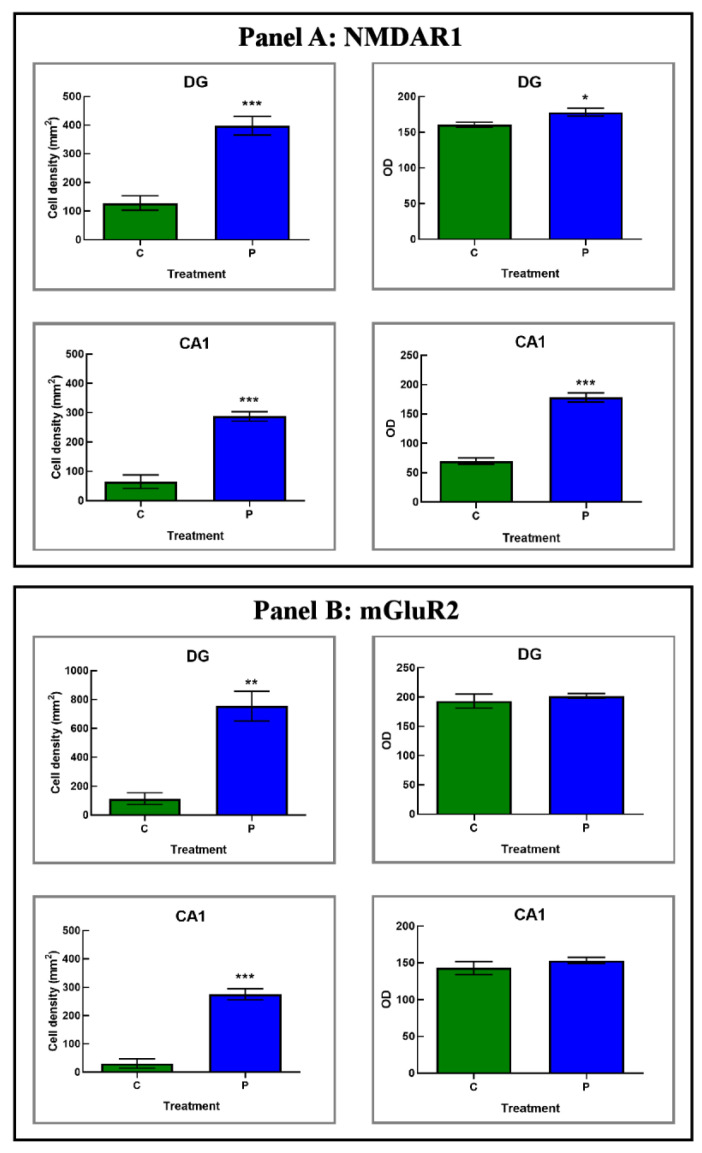
Panels (**A**,**B**): histograms displaying the quantitative evaluation of NMDAR1- and mGluR2-immunopositive cell density and OD, respectively, measured in DG and CA1 area of C and P mice. p values calculated by unpaired Student’s *t*-test: *p* < 0.05 (*), *p* < 0.01 (**), and *p* < 0.001 (***).

Interestingly, we revealed an increase in NMDAR1-immunopositivity, both in the DG and the CA1 region of the Ammon’s horn of P mice, compared to controls (Figure 8 and Figure 9 and Table 5). Specifically, in P animals only the heaviest immunoreactivity was clearly detected in the DG where clusters of strikingly immunopositive cells, arranged in well-ordered chains, were observed in the width of the GL (Figure 8). Noticeably, several immunolabelled cells were also located in the SGZ, and many immunoreactive neurons, characterized by large soma, were discerned in the PL (Figure 8). As regards the CA1 area, the SP exhibited several NMDAR1-immunopositive neurons, somewhere showing palely immunomarked tiny prolongations, deepening in the underneath SR (Figure 8).

Based on the subsequent quantitative evaluation, NMDAR1-immunoreactivity, considered in terms of both cell density and OD, significantly enhanced in P mice compared to C animals in both considered areas with the stronger effect measured in the CA1 region. In detail, considering the DG, an extremely significant enhancement of immunolabeled cell density was reported in P mice compared to controls (Figure 9 and Table 5). Further, in the same region, a significant increase in NMDAR1-immunopositive cell OD was determined comparing P and C animals (Figure 9 and Table 5). Displaying a similar trend, even with more pronounced effects, the NMDAR1-immunoreactivity, evaluated in terms of both cell density and OD, significantly enhanced in the CA1 area of P mice compared to controls (Figure 9 and Table 5).

With regard to mGluR2, its cellular localization and distribution bared an extensive spreading both in the DG and the CA1 region of the Ammon’s horn of P mice compared to controls (Figure 8 and Figure 9 and Table 5). Control mice showed a weaker and much less widespread immunoreactivity, thus revealing an immunostaining pattern analogous to that described above for the ionotropic receptor NMDAR1. In particular, in P mice the DG was characterized by some heavily mGluR2-immunopositive cells localized both in the width of the GL as well as close to the SGZ. Additionally, in the same area some mGluR2-immunoreactive large neurons were noticeable in the PL (Figure 8). Considering the CA1 region in P animals, several mGluR2-immunopositive neurons were discernible in the SP, frequently characterized by immunoreactive prolongations, deepening beneath in the underlying SR (Figure 8).

Notably, comparing P mice with controls, the quantitative analysis documented a significant increase in mGluR2-immunoreactive cell density in both considered areas, i.e., DG and CA1, with the latter region showing the most striking effect (Figure 9 and Table 5). Diversely, focusing on mGluR2-immunopositive cell OD, any significant difference was measured neither in the DG nor in the CA1 area comparing P and C animals (Figure 9 and Table 5).

## 4. Discussion

Hippocampus is one of the most vulnerable organs of the CNS to neurodegeneration during aging and inflammation, and the contiguous and interconnected regions of hippocampus show different, region-specific, reactivity to aging [54].

He2 primordium extract contains a pure amount of ERGO, a powerful antioxidant, about three times higher compared to that measured in previously described extracts [7,41]. In a previous paper we tested the preventive effect of this primordium extract on locomotor frailty during normal aging and its antioxidants profile on cerebellar cortex [6].

Currently, with the aim of assessing the preventive effect of ERGO on cognitive frailty development during aging, we performed a longitudinal study monitoring the cognitive decline both in the knowledge and the remember components of the recognition memory. We focused on the consequences for in vivo recognition memory during physiological aging, and in parallel demonstrating changes in CA1 and DG regions on different pathways, crucially linked to this cognitive function, i.e., inflammation, oxidative stress and glutamatergic neurotransmission.

ERGO-rich He2 primordium was administered to mice starting from 15 months of age, during the adulthood life phase, until 23 months of age, corresponding to the senescence period. In search of a translational approach “from bench to bedside”, we intentionally chose the dose of 1 mg/day to mirror the usual human oral intake (1 g/day) and we chose spontaneous behavioral tests resembling those used in clinical practice. All reported results were obtained in age-matched supplemented and non-supplemented control mice.

**Figure 10:**
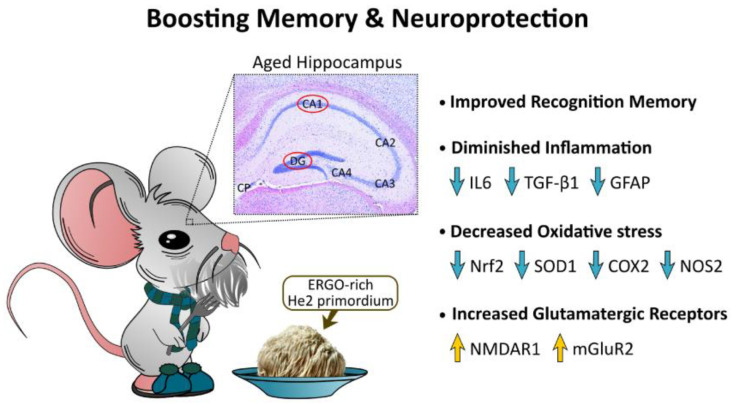
Pictographic drawing summarizing main findings and *take-home* message.

Wide consensus among scientists agrees that recognition memory is composed of two components: (i) the knowledge/familiarity component; and (ii) the remember/recollection component, which can be accessed via the use of different spontaneous object exploration paradigms. During aging, the two components of recognition memory declined in a different manner. Notably, the preventive effect of ERGO on the physiological age-related decline of the two components was different: the knowledge frailty index increased less than in non-supplemented mice, whereas the remember component reached a “gain of function” even compared to young animals (Figure 10). A debate is still open regarding the role of the medial temporal area of the brain in the two components of the recognition memory. The so-called “unitary strength model” theory asserts that recognition memory could be a unitary declarative system recognizing different strength in memory traces. Diversely, the so-called “dual-process model” theory postulates that recollection and familiarity are placed in anatomically and functionally distinct regions in medial temporal brain areas. Our previous in vivo investigations demonstrated a differential effect on the two described components of the recognition memory. In agreement with our previous data of He1 extracts, the differential effect of this ERGO-rich extract supports the “dual process model” theory [42,43].

Furthermore, we cannot exclude that other brain areas involved in the recognition memory through a multisynaptic loop [55], and less vulnerable to aging process, could be differently exploited by young and aged mice. As recently published, young rats predominately used hippocampal solutions while old rats employed striatal solutions on different recognition memory tasks. In particular, it was described as an enhanced performance on a striatal object replacement task over time with increasing aging [56].

Cytokine dysregulation deeply affects the remodeling of the immune system in the elderly, triggering to uncontrolled systemic inflammation, the so-called *inflammaging*, a typical hallmark of unsuccessful aging. For instance, high levels of IL6 are associated in the aged subject with increased risk of morbidity and mortality [57,58].

As regards inflammation, we currently revealed a significant lessening of both IL6 and TGFβ1 in hippocampus of supplemented mice. In detail, IL6 significantly diminished in both the DG and CA1 (mainly in SP layer) of P animals, while TGFβ1 levels decreased in the DG region only (predominantly in GL and POL layers). In parallel, we unveiled a marked reduction in GFAP in hippocampus of P mice, both in DG and CA1 regions, thus supporting the reduction in the age-related reactive gliosis typically observed in physiological aging in non-supplemented animals (Figure 10). These data are in line with the notion that reactive gliosis during aging could be caused by an increased number and/or activity of astrocytes [59].

Concerning oxidative stress pathway, Nrf2 plays different key roles depending on ongoing oxidative conditions. This crucial pleiotropic transcription factor, pivotally implicated in aging and lifespan regulation, is able to both up- and down-regulate the expression of several gene and enzymes, also boosting the maintenance of cellular resistance against oxidants [17,60,61]. Our findings demonstrated a decrease in Nrf2 expression levels both in the CA1 area, as well as in the DG (mainly closed to SGZ and in POL) of P mice compared to the same regions of age-matched not supplemented animals (Figure 10).

Several investigations also explored age-related changes in SOD activity, but often the reported results are inconsistent, or even contradictory. In fact, in vivo studies showed an age-dependent decrease in SOD1 activity in the brains of old rats. Differently, other investigations even reported that SOD1 activity increased with aging in some tissues such as mouse skeletal muscles and brain [62,63]. Therefore, it remains elusive which mechanism may cause the controversy over age-related changes in SOD1 activity. Presently, we revealed a significant lessening in the SOD1 expression levels in supplemented mice in the DG only, with the CA1 remaining unaffected (Figure 10).

The oxidative/nitrosative imbalance, characterized by continuous ROS generation as a typical hallmark of an age-related persistent condition of oxidative stress, is able to provoke chronic inflammation through lipid peroxidation and pro-inflammatory cytokines enhancement [64,65]. In particular, the excessive NO production was described in numerous pathological conditions such as neurodegenerative diseases, *inflammaging*, and ischemia [66]. Our current findings proved a significant selective diminishing of both NOS2 and COX2 expression levels in the CA1 area (Figure 10). This expression pattern, immunohistochemically disclosed in the hippocampus, is similar to that previously observed in the cerebellum of aged mice fed with the same ERGO-rich supplementation. Further, our current findings are in line with previous literature revealing that natural extract-enriched diet diminished age-associated increased expression of COX2 and NOS2 [67]. It should also be mentioned that enzymatic sources of oxidative mediators in the brain are controlled by the glutamatergic NMDARs activation, suggesting that excitatory amino acids determined stress-induced NOS2 and COX2 expression/activity in the brain [67,68].

The glutamatergic pathway is crucially involved in learning, memory formation/storage and synaptic plasticity [23]. Several studies in pre-clinical models revealed during aging a decrease in the number of glutamatergic neurons [69], a decline in glutamate level in the hippocampus [5] paralleled by hippocampal detrimental changes in synaptic composition and function [70]. It has also been demonstrated that a disrupted glutamate balance guides to the perturbation of glutamate neurotransmission which may bring severe consequences, possibly leading to the onset of dementia and neurodegenerative diseases [71,72,73,74].

Glutamatergic receptors, both ionotropic and metabotropic, keep the ability to induce synaptic plasticity in the hippocampus [75]. In murine models the characteristic high-density amount of NMDAR1 in the hippocampus plays a key role in the initiation steps of learning and memory [5,76]. In fact, NMDA receptors are known to be pivotally involved in the performance of many different memory tasks including those using spatial, reference, working and passive avoidance memory, being also essential to LTP in many different brain regions [77,78]. A wide scientific consensus exists that NMDARs declined both in binding densities as well as in functions with increasing age, also differing the NMDARs subunit compositions in the aged individuals [5]. Furthermore, behavioral and electrophysiological evidence supported the role of NMDARs [51] and G protein coupled metabotropic receptors in neural plasticity changes with aging [26,30,31]. In the current study we revealed that ERGO-rich He2 primordium was strikingly effective to improve NMDAR1 expression levels (in terms of both cell density and OD) in the hippocampus of aged mice, both in the DG and CA1. In a similar manner, after the eight-month He2 oral supplementation a marked enhancement of mGluR2-immunoreactive cell density in the same hippocampal regions was revealed in aged mice (Figure 10). Based on these findings, we postulated that ERGO-rich He2 primordium extract was able to better glutamatergic pathway/neurotransmission in aged mice.

## 5. Conclusions

In conclusion, our findings demonstrated the functional neuroprotective role of an eight-month lasting oral supplementation with He2 primordium in preventing cognitive deterioration (Figure 10). A bulk of literature data demonstrated in vivo the beneficial effects of ERGO on learning, cognition, and memory using different models of age-related and neurodegenerative diseases [79,80,81,82,83]. Based on all these data we reasonably supposed that the nootropic described effects could be ascribable to ERGO, since the well-known erinacines and hericenones were not present [40], although we cannot exclude the neuroprotective effects due to other metabolites. The recovery and boosting of both components of the recognition memory were paralleled by a neuroprotective action on hippocampus that was characterized by a lessening of inflammation and oxidative stress accompanied by an increase in ionotropic and metabotropic glutamate receptors expression. In different hippocampal areas, ERGO-rich He2 primordium elicited selective changes in the explored biomarkers.

As a concern to inflammation, the supplementation protects both DG and CA1 regions leading to a diminished IL6 production, with a measured 7-8-time folder lessening of IL6-immunopositive cell density. Diversely, only the DG displayed a significant decrease in TGFbeta1 and GFAP in P mice, while the CA1 region appeared unaffected (Figure 10). Therefore, we highlighted a selective preventive and neuroprotective efficacy of ERGO-rich He2 primordium against *inflammaging* process in the DG.

Similarly, ERGO-rich He2 primordium displayed a differential selective preventive and neuroprotective effect against oxidative stress on the two hippocampal areas, i.e., DG and Ca1. Specifically, the DG of P mice was characterized by reduced Nrf2 and SOD1 levels (about three and four times lower, respectively, compared to those measured in C animals), with NOS2 and COX2 remaining unaffected. Differently, in the CA1 area we proved an ERGO-rich He2 primordium-induced significant diminishing of NOS2 and COX2 (about three and 10 times lower, respectively, compared to those assessed in C mice), paralleled by a lessening of Nrf2 levels without any changes in SOD1 expression levels (Figure 10).

Furthermore, our data indicated that ERGO-rich He2 primordium was effective to lead to an increase in NMDAR1 and mGluR2 glutamatergic receptors, both in CA1 and DG regions (Figure 10).

Taken as a whole, these data led us to assume that as these molecules are pivotally involved in age-associated damages the assessed expression changes induced by ERGO-rich He2 primordium could feasibly boost lifetime, or even better life quality. Strikingly, it must be emphasized that the reported modifications in the expression patterns of these key molecules were accompanied by the amelioration of cognitive performances.

## Figures and Tables

**Table 1:** Selected parameters for studying cognitive (Emergence, NOR, OL, Y maze) and locomotor (Open arena) performances of mice at four selected experimental time.

Spontaneous Behavioral Test	Selected Cognitive Parameters
Emergence	Exit Number (n)
	Latency of First Exit (s)
	Time of Exploration (s)
NOR	Number of Approaches: DI
	Time of Approaches: DI
OL	Number of Approaches: DI
	Time of Approaches: DI
Y maze	Alternation %

**Table 2:** Primary and secondary antibodies.

	Antigen	Immunogen	Manufacturer, Species, Mono-Polyclonal, Cat./Lot. No., RRID	Dilution
**Primary** **antibodies**	Anti-Interleukin-6 (M-19)	Purified antibody raised against a peptide mapping at the C-terminus of murine IL6	Santa Cruz Biotechnology (Santa Cruz, CA, USA), Goat polyclonal IgG, Cat# sc-1265, RRID: AB_2127470	1:100
	Anti-Transforming Growth Factor β1 (V)	Purified antibody raised against a peptide mapping at the C-terminus of TGF-β1 of human origin	Santa Cruz Biotechnology (Santa Cruz, CA, USA), Rabbit polyclonal IgG, Cat# sc-146, RRID: AB_632486	1:100
	Anti-Glial fibrillary acidic protein (C-19)	Purified antibody raised against a peptide mapping at the C-terminus of GFAP of human origin	Santa Cruz Biotechnology (Santa Cruz, CA, USA), Goat polyclonal IgG, Cat# sc-6170, RRID: AB_641021	1:100
	Anti-Nuclear factor erythroid 2–related factor 2	Purified antibody raised against a peptide within Human Nrf2 aa 550 to the C-terminus	Abcam (Cambridge, UK), Rabbit polyclonal IgG, Cat# ab31163, RRID: AB_881705	1:100
	Anti-Superoxide Dismutase-1 (FL-154)	Purified antibody raised against amino acids 1–154 representing full-length human SOD-1	Santa Cruz Biotechnology (Santa Cruz, CA, USA), Rabbit polyclonal IgG, Cat# sc-11407, RRID: AB_2193779	1:100
	Anti- Cyclooxygenase-2 (M-19)	Purified antibody raised against a peptide mapping at the C-terminus of COX2 of mouse origin	Santa Cruz Biotechnology (Santa Cruz, CA, USA), Goat polyclonal IgG, Cat# sc-1747, RRID: AB_2084976	1:100
	Anti-Nitric Oxide Synthases-2 (M19)	Purified antibody raised against a peptide mapping at the C-terminus of NOS2 of mouse origin	Santa Cruz Biotechnology (Santa Cruz, CA, USA), Rabbit polyclonal IgG, Cat# sc-650, RRID: AB_631831	1:100
	Anti- N-methyl-D-aspartate Receptors 1	Purified antibody raised against a peptide corresponding to the C-terminus of rat NMDA receptor subunit	Millipore—Merck KGaA (Darmstadt, Germany), Rabbit monoclonal IgG, Cat# AB9864, RRID: AB_2112158	1:500
	Anti-Glutamate Receptor 2 and 3	Purified antibody raised against a peptide mapping at the C-terminus of rat GluR2	Millipore—Merck KGaA (Darmstadt, Germany), Rabbit polyclonal IgG, Cat# AB1506, RRID: AB_ 90710	1:100
**Secondary** **Antibodies**	Biotinylated goat anti-rabbit IgG	Gamma immunoglobulin	Vector Laboratories (Burlingame, CA, USA), Goat, lot# PK-6101, RRID: AB_2336820	1:200
	Biotinylated rabbit anti-goat IgG	Gamma immunoglobulin	Vector Laboratories (Burlingame, CA, USA), Rabbit, Cat# PK-6105, RRID: AB_2336824	1:200

**Table 3:** Quantitative measurement of inflammation markers, namely IL6, TGFβ1 and GFAP in the hippocampus, precisely in the DG and CA1 of C and P mice. n.c.: not comparable.

		IL6		TGFbeta1		GFAP	
		Cell Density	OD	Cell Density	OD	Cell Density	OD
**DG**	C	310.79 ± 17.86	178.50 ± 9.40	728.24 ± 33.81	142.20 ± 2.96	298.24 ± 19.37	137.69 ± 17.17
	P	42.26 ± 6.27	120.00 ± 4.21	119.01 ± 35.41	121.06 ± 4.62	165.69 ± 12.69	86.78 ± 4.49
**CA1**	C	289.13 ± 19.61	127.80 ± 6.63	n.c.	30.49 ± 1.44	109.87 ± 13.47	163.84 ± 3.68
	P	44.48 ± 21.82	96.68 ± 3.47	n.c.	30.97 ± 0.56	89.71 ± 7.53	138.49 ± 1.55

**Table 4:** Quantitative assessment of Oxidative stress key molecules, namely Nrf2, SOD1, COX2, and NOS2, in the DG and CA1 of C and P mice. n.c.: not comparable.

		Nrf2		SOD1	
		Cell Density	OD	Cell Density	OD
**DG**	C	258.84 ± 14.41	169.78 ± 13.00	415.97 ± 32.21	140.49 ± 7.22
	P	79.24 ± 10.42	125.38 ± 9.66	98.74 ± −12.40	111.20 ± 3.67
**CA1**	C	341.02 ± 41.94	139.38 ± 1.37	n.c.	45.68 ± 2.70
	P	66.72 ± 23.05	82.01 ± 4.72	n.c.	45.19 ± 2.03
		**COX2**		**NOS2**	
		**Cell Density**	**OD**	**Cell Density**	**OD**
**DG**	C	241.23 ± 18.33	100.20 ± 2.58	30.52 ± 2.32	90.91 ± 12.17
	P	192.81 ± 14.11	100.01 ± 3.23	29.63 ± 2.69	89.84 ± 2.97
**CA1**	C	404.04 ± 49.32	119.39 ± 9.47	253.78 ± 38.06	123.79 ± 7.98
	P	40.77 ± 16.44	82.39 ± 2.65	70.78 ± 25.40	67.95 ± 2.18

**Table 5:** Quantitative appraisal of glutamatergic neurotransmission indicators, namely NMDAR1 and mGluR2, in hippocampal areas, i.e., DG and CA1, in C and P mice.

		NMDAR1		mGluR2	
		Cell Density	OD	Cell Density	OD
**DG**	C	127.78 ± 25.13	160.44 ± 3.59	113.08 ± 41.06	193.14 ± 12.01
	P	397.74 ± 32.78	178.13 ± 5.42	753.86 ± 102.81	201.83 ± 4.28
**CA1**	C	64.26 ± 23.40	69.65 ± 5.44	30.15 ± 16.29	142.90 ± 8.70
	P	287.26 ± 16.33	177.71 ± 7.80	275.16 ± 19.83	153.10 ± 4.18

## Data Availability

Not applicable.
